# Human papillomavirus prevalence among indigenous and non-indigenous Australian women prior to a national HPV vaccination program

**DOI:** 10.1186/1741-7015-9-104

**Published:** 2011-09-13

**Authors:** Suzanne M Garland, Julia ML Brotherton, John R Condon, Peter B McIntyre, Matthew P Stevens, David W Smith, Sepehr N Tabrizi

**Affiliations:** 1 Regional World Health Organisation Human Papillomavirus Laboratory Network, Department of Microbiology and Infectious Diseases, The Royal Women's Hospital, Locked Bag 300, Parkville, Victoria 3052, Australia; 2 Department of Obstetrics and Gynaecology, University of Melbourne, Parkville, Victoria 3052, Australia; 3 Department of Microbiology, Royal Children's Hospital, Locked Bag 300, Parkville, Victoria 3052, Australia; 4 Murdoch Childrens Research Institute, Parkville, Victoria 3052, Australia; 5 National Centre for Immunisation Research and Surveillance, University of Sydney and The Children's Hospital at Westmead, Locked Bag 4001, Westmead, NSW 2145, Australia; 6 Registries, Victorian Cytology Service, PO Box 310, East Melbourne, Victoria 8002, Australia; 7 Menzies School of Health Research, Box 41096, Casuarina, Northern Territory 0811, Australia; 8 PathWest Laboratory Medicine WA, Locked Bag 2009, Nedlands, Western Australia 6909, Australia

## Abstract

**Background:**

Indigenous women in Australia have a disproportionate burden of cervical cancer despite a national cervical screening program. Prior to introduction of a national human papilloma virus (HPV) vaccination program, we determined HPV genotype prevalence by Indigenous status and residence in remote areas.

**Methods:**

We recruited women aged 17 to 40 years presenting to community-based primary health services for routine Pap screening across Australia. A liquid-based cytology (LBC) cervical specimen was tested for HPV DNA using the AMPLICOR HPV-DNA test and a PGMY09/11-based HPV consensus PCR; positive specimens were typed by reverse hybridization. We calculated age-adjusted prevalence by weighting to relevant population data, and determined predictors of HPV-DNA positivity by age, Indigenous status and area of residence using logistic regression.

**Results:**

Of 2152 women (655 Indigenous), prevalence of the high-risk HPV genotypes was similar for Indigenous and non-Indigenous women (HPV 16 was 9.4% and 10.5%, respectively; HPV 18 was 4.1% and 3.8%, respectively), and did not differ by age group. In younger age groups, the prevalence of other genotypes also did not differ, but in those aged 31 to 40 years, HPV prevalence was higher for Indigenous women (35% versus 22.5%; *P *< 0.001), specifically HPV clades α5 (OR = 2.1, 95% CI 1.1 to 4.3) and α7, excluding type 18 (OR 1.9, 95% CI 1.1 to 3.3). In multivariate analysis, detection of any HPV genotype was strongly associated with smoking and Pap-test abnormalities, with both risk factors more common among Indigenous women.

**Conclusion:**

Although we found no difference in the prevalence of HPV16/18 among Australian women by Indigenous status or, for Indigenous women, residence in remote regions, differences were found in the prevalence of risk factors and some other HPV genotypes. This reinforces the importance of cervical screening as a complement to vaccination for all women, and the value of baseline data on HPV genotype prevalence by Indigenous status and residence for the monitoring of vaccine impact.

## Background

Since the introduction of a comprehensive, organized, cervical cytology (Pap) screening program in 1991, Australia has seen a marked reduction in incidence of cervical cancer from 13.2 per 100,000 in the early 1990s to a current stable rate of around 6.9 per 100,000 since the early 2000s. This has been matched by a mortality reduction from 4 to 1.9 per 100,000 for the respective time periods[1]. However, there are large disparities within the total Australia population; incidence and mortality are higher for regional and remote than urban residents, and much higher (approximately two and five times higher respectively) for Indigenous (Aboriginal and Torres Strait Islander women) than other Australian women [1].

Cervical cancer is caused by oncogenic human papillomavirus (HPV) types,[2] with 70% of cases due to HPV 16 and 18 genotypes both worldwide and in Australia[3]. Given the early results of Phase 3 vaccine trials of a bivalent [4] and quadrivalent vaccine[5,6], which have now shown efficacy against HPV infection and related cervical disease, safety and immunogenicity, we embarked on a study (the Women's HPV Indigenous Non-Indigenous Urban Rural Study; WHINURS) to compare the prevalence of genotype-specific HPV among Indigenous and non-Indigenous women residing in both remote and urban settings across Australia. The primary aim was to establish whether there was any substantive difference in the prevalence of specific HPV genotypes, particularly vaccine-preventable genotypes, prior to an HPV vaccination program, by region of residence and Indigenous status. This was based on precedents in which such differences were present for other infectious diseases [7-10] in these groups, as well as some limited data from small surveys suggesting similar differences for HPV[11].

Existing Australian data on cervical HPV prevalence in the general population are limited, with either none or, in one case, only small study datasets from subpopulations included in international meta-analyses of prevalence in women with low-grade or normal cytology[12-15]. Although these international data have indicated that HPV16 is the most common HPV type detected across the world, differences in the prevalence of the next most common types have been noted between regions. Few studies have been conducted to date directly evaluating HPV types in Indigenous populations compared with non-Indigenous populations. This is particularly important given that Indigenous in many developed countries that are already deploying HPV vaccines, such as the US, Canada, Australia and New Zealand, have high rates of cervical cancer. Should the prevalence of vaccine-preventable HPV types in Indigenous women differ from those of non-Indigenous women, then there is a risk that vaccination could widen rather than reduce existing inequities in cervical cancer. This is further compounded by the current lack of knowledge as to whether non-vaccine types may become more prevalent (both in absolute and relative terms) after vaccination. Only one study has previously sought to determine possible differences between Indigenous and non-Indigenous Australian women in prevalence of specific HPV types[16]. Using tampon-collected specimens and an L1-based PCR system capable of typing 10 HPV types, that study found that non-Indigenous women had higher rates of HPV detected (56% versus 42%): however the analysis did not account for differences between the two groups in parameters such as sites of recruitment, cytology results or age.

In April 2007, Australia commenced the most comprehensive government-funded national HPV vaccination program in the world, using the quadrivalent vaccine for all girls 12-13 years of age as an ongoing program, with a catch-up program delivered through school and community providers for women up to 26 years of age, which finished on 31 December 2009. The data from this study provide an important baseline for comparisons internationally by epidemiologic setting and ethnicity before and after the commencement of widespread programs of HPV vaccination.

## Methods

The aim of this study was to determine whether there were any differences in HPV type prevalence by area of residence or ethnicity, and this aim guided our choice of methods. Engagement and recruitment of Indigenous women in remote areas, by providing the opportunity to participate meaningfully in improving their own health and that of their communities, was recognized as crucial to the success of the study. Therefore, the sampling strategy was guided by the use of methods that would include these women in a culturally appropriate way.

### Consultation, ethics, pilot site, and development of culturally appropriate materials

In recognition of the need for research to benefit Indigenous communities, and for Indigenous people to be involved in the design, planning, undertaking and dissemination of Indigenous research, we identified collaborative research partners involved in frontline community care and service provision in the area of cervical cancer prevention in Indigenous communities. Prior to initiation of the study, we undertook consultation with Indigenous communities (including the establishment of an Indigenous Steering Committee), medical services, healthcare workers, public-health practitioners and servicing cytology groups, commencing in January 2005, including on-site workshops and subsequent staff training. Protocols and communication materials (including flipcharts and brochures) were developed to support the study, with local input at different sites to tailor the materials so that they would be locally and culturally appropriate as needed. A pilot study was conducted in central Australia between April and July 2005 to examine feasibility, communication materials and protocols, and laboratory techniques: it was endorsed by the Central Australian Human Research Ethics Committee. The multisite study was approved by 34 site ethics committees. Women were enrolled into the study between July 2005 and February 2008.

### Study population, recruitment methods, consent and specimen collection

Women aged 18 to 40 years who were attending their usual healthcare provider for routine Pap smear cytology were invited to participate in the study. Some women aged under 18 years, who were assessed as mature minors and hence competent for giving consent without parental or guardian involvement and attending for a routine Pap test, were also enrolled by providers. Although most women in Australia see a general practitioner (primary-healthcare providers) for Pap-test screening, our primary focus was upon adequate recruitment of Indigenous women, and therefore we targeted Indigenous health providers and clinics, and equivalent service providers (community-based, no fee for service) for non-Indigenous women, to ensure comparability of Indigenous and non-Indigenous women in the study. The study sample size was determined based on the HPV prevalence estimates from a large community-based study in the USA of women aged 18-40 attending for a Pap smear and was powered (α = 0.05, 1-β = 0.90) to detect an absolute difference in HPV16/18 of ≤ +/-5% between Indigenous and non-Indigenous women, and between Indigenous women residing in remote areas and other Indigenous women[17]. We determined the sample size necessary at each site according to the predominant client population of the site (Indigenous or non-Indigenous) and the remoteness category of the site according to the Accessibility/Remoteness Index of Australia (ARIA). The ARIA assigns a 'remoteness score' based on each locality's relative access to services, which is estimated by road distance (or other transport mechanism for islands) to the nearest population centers of several different sizes. These scores are grouped into five categories: major cities, inner regional, outer regional, remote, and very remote. Approximately two-thirds of the Australian population [18], but only one-third of the Indigenous population, live in major cities [19]

We recruited women in all states and one territory (Northern Territory) of Australia from sixteen Indigenous health services, eight family-planning services and ten community clinics. Owing to the incorporation of the study into routine clinical care, response rates (number enrolled divided by number approached) were not accurately recorded at all sites. Where response rates were recorded or estimated from the number of eligible (by age) women who had a Pap test taken during the recruitment period, these ranged from 46% to 87%. Recruitment of Indigenous women in some sites was challenging, and required further strategies for community engagement with the study to be developed[20,21].

Women were asked to allow the cervical samples collected for Pap cervical cytology to be tested for HPV DNA also. Once fully informed consent was obtained, Pap smear samples were collected in the routine clinic settings. In addition to routine pathology specimen details, information confirming Indigenous status, most recent previous Pap-test result, pregnancy status, smoking status and hormonal contraceptive use was requested from participants. Following the testing of specimens, identifying details were used to report Pap cytology and high-risk (HR) HPV DNA results back to the clinician.

Most specimens were collected using a brush (Cervex; Rovers Medical Devices BV, The Netherlands) and a spatula. The sample was then smeared onto a glass slide and fixed with ethanol ready for conventional Pap cytology, and the remainder was rinsed into vials containing fixative solution (ThinPrep vials with PreservCyt fixative; Cytyc Corp., Boxborough, MA, USA). In a minority of samples, LBC was standard, and hence an aliquot was removed using good microbiological practices within a biohazard cabinet, prior to processing for cytology. All samples were transported to the Regional WHO LabNet for HPV located at the Royal Women's Hospital in Melbourne, Australia, for HPV DNA detection and genotyping.

### Algorithm for feedback of results and follow-up by clinicians

Results of the HR HPV testing were returned to the woman's healthcare provider so that they could be conveyed back by trained staff to each participant in a culturally appropriate way. All women were managed according to National Health and Medical Research Council guidelines for the follow-up of abnormal Pap tests[22]. Currently, HPV DNA testing in clinical practice in Australia is only recommended as a "test-of-cure" following local ablative or excisional therapy for precursor cervical cancer lesions of high-grade (2+) cervical intraepithelial neoplasia (CIN)[22].

### HPV DNA detection and genotyping

DNA was extracted from stored specimens (in PreservCyt) using a commercial isolation and purification system (MagNA Pure LC; Roche Molecular Systems, Alameda, CA, USAusing 1-ml aliquots of the preserved specimens as previously described[23,24]. From a final volume of 100 μl of resultant DNA, 10 μl of extracted DNA was screened (AMPLICOR HPV test; Roche Molecular Systems) for detection of 13 HR HPV genotypes (types 16, 18, 31, 33, 35, 39, 45, 51, 52, 56, 58, 59 and 68). As this test detects only 13 HR HPV types, all samples testing negative for HR HPV by this test were also tested to exclude presence of other types, using a 20 μl aliquot of extracted DNA in a PGMY09/11-based HPV consensus PCR assay[25]. A PCR-ELISA detection protocol was used, as described previously[26]. All assays used incorporated the amplification of the *β-globin *gene as an internal control.

All samples that tested positive by the AMPLICOR or PGMY09/11 PCR test were genotyped (HPV Linear Array^® ^(LA) Genotyping Test; Roche Molecular Systems), using 50 μl of extracted DNA, and following the manufacturer's instructions with minor modifications as previously reported[24,27,28]. LA identifies 37 genotypes: 6, 11, 16, 18, 26, 31, 33, 35, 39, 40, 42, 45, 51, 52, 53, 54, 55, 56, 58, 59, 61, 62, 64, 66, 67, 68, 69, 70, 71, 72, 73, 81, 82 (previously known as IS39), 83, 84 and 89 (previously known as CP6108) [29]. There were 13 specimens that were weakly positive only to the PGMY09/11 PCR but negative on LA, and these specimens were classified as HPV negative.

Specimen contamination and carryover were prevented by use of barrier tips, prior division of all reagents into aliquots, and performance of pre- and post-PCR steps in different rooms specifically allocated for PCR. Negative and positive controls were processed with each run, and lack of signal in the negative control was used to monitor possible carryover.

### Statistical analysis

The ARIA category was assigned using the postcode of each participant's residence. Pap-test results were reported by cytology laboratories (usual service providers) according to the Australian Modified Bethesda classification, 2004 [22]. For the purposes of analysis, 'possible' low-grade squamous intraepithelial lesion (LSIL) Pap results were grouped LSIL and 'possible' high-grade squamous intraepithelial lesion (HSIL) Pap results were grouped with HSIL.

Age-adjusted prevalences were calculated by weighting the WHINURS sample to the relevant Australian Bureau of Statistics (ABS) population structure, by single year of age. Data were analyzed using SPSS software (version 17.0; SPSS Inc., Chicago, IL, USA), except for weighted confidence intervals, which were calculated using STATA software (StataCorp LP, College Station, TX, USA). Univariate analysis compared proportions using Pearson's χ^2 ^tests (or Fisher's exact test when the expected count in any cell was < 5), and mean ages using *t*-test. Binary logistic regression was used for multivariate analysis. Interaction terms were created for predictor variables to check for effect modification. No significant interactions were identified. Because of the high correlation between living in a remote area and Indigenous status, the effect of area of residence was analyzed separately for the two groups of women, with the analysis of non-Indigenous women comparing residence in a major city versus non-major city.

Indigenous status and area of residence were variables of prior interest. Multivariate models also included other known determinants of HPV infection: age, smoking status, and use of hormonal contraception. We did not collect sexual history from participants. Although some women were pregnant, there was no association between pregnancy and HPV detection in univariate analysis, and hence this was not considered further.

## Results

Of a total of 2156 samples from women age 15 to 40 years, four were found to have no ß-globin signal, indicating inadequate sample collection, and these were excluded from the analysis, leaving 2152 participants with HPV results available (655 Indigenous, 1494 non-Indigenous, 3 unknown ethnicity) (Table 1). Half of the Indigenous participants were recruited from remote or very remote areas, compared with 6% of the non-Indigenous participants.

**Table 1:** Demographic characteristics of WHINURS^a ^participants aged ≤40 years, by Indigenous status^b^

Variable	Non-Indigenous		Indigenous	
	
	n	%	n	%
Participants	1494		655	
Age, years				
15 to 20	139	9.3%	117	17.9%
21 to 25	359	24.0%	178	27.2%
26 to 30	383	25.6%	137	20.9%
31 to 35	341	22.8%	124	18.9%
36 to 40	272	18.2%	99	15.1%
Median	29		27	
Mean^c^	28.9		27.4	
Hormonal contraception user^c,d^	643	43.3%	196	30.3%
Smoker^c,e^	368	24.8%	363	56.2%
Pregnant^c,f^	13	0.9%	54	8.2%
Remoteness^g^				
Major cities	868	58.1%	45	6.9%
Inner regional	182	12.2%	65	9.9%
Outer regional	351	23.5%	215	32.8%
Remote	78	5.2%	135	20.6%
Very remote	15	1.0%	195	29.8%
Most recent Pap-test result^b^				
Normal	1143	81.2%	453	77.3%
Low-grade abnormality	116	8.2%	44	7.5%
High-grade abnormality	5	0.4%	7	1.2%
First Pap	144	10.2%	82	14.0%
Unknown/missing/unsatisfactory	19/45/22		21/42/6	
Current Pap-test result^b,f^				
Normal	1296	88.0%	527	84.7%
Low-grade abnormality	156	10.6%	70	11.3%
High-grade abnormality^b^	20	1.4%	25	4.0%
Unsatisfactory	20		7	

^a^Women's HPV Indigenous Non-Indigenous Urban Rural Study

^b^Three participants with unknown Indigenous status are not included in this table

^b^*P *< 0.001 for group Indigenous women versus non-Indigenous women.

^c^Data missing for ten non-Indigenous and eight Indigenous participants.

^d^Data missing for ten non-Indigenous and nine Indigenous participants.

^e^Data missing for eight non-Indigenous and six Indigenous participants.

^f^Data missing for two non-Indigenous and twenty-six Indigenous participants.

^g^Remote residence refers to residence in an area categorized as remote or very remote using the Accessibility/Remoteness Index of Australia classification by postcode. Non-remote residence refers to areas categorized as major cities or regional areas.

### Characteristics of participants

Regarding demographic variables, Indigenous women were more likely to be younger, to smoke, to be pregnant and less likely to use hormonal contraception (Table 1). Regarding Pap-test-related variables, a higher proportion of Indigenous than non-Indigenous women were having their first ever Pap test (13.8% versus 10.1%; *P *= 0.02), and the prevalence of abnormal Pap-test results was higher. The prevalence of HSIL as predicted by cytology was higher for Indigenous than non-Indigenous women for both their most recent previous Pap test (1.4% versus 0.4%, *P *= 0.02), and for the Pap test performed during this study (current Pap test) (4.0% versus 1.4%, *P *< 0.001). Of the 45 women with a current high-grade abnormal Pap test, 25 (56%) were Indigenous, including one with squamous cell carcinoma and one with possible microinvasive cancer.

Demographic differences between Indigenous women from remote compared with non-remote areas were small and not significant, with one exception: a higher proportion of women from remote areas had normal results on their most recent previous Pap test compared with women from non-remote areas (93.8% versus 85.8% of women for whom the previous Pap result was known, *P *= 0.003) (Table 1). To evaluate the representativeness of our sample, we compared the prevalence of abnormal Pap tests in non-Indigenous study participants with data from the Victorian Cervical Cytology Registry (which services women resident in the state of Victoria, Australia, representing a population of 2.7 million women) on the age-adjusted prevalence of abnormal Pap tests among resident women aged 15-39 (national data were not available). The age-adjusted prevalences for the study population and for women in Victoria were similar: respectively, 88.2% and 88.9% for normal Pap tests; 10.4% and 9.5% for LSIL; and 1.3% and 1.7% for HSIL/cancer.

### Cervical HPV DNA prevalence

The age-adjusted prevalence of detection of any HPV DNA was higher for Indigenous than for non-Indigenous women when standardized to the general Australian population, although confidence intervals overlapped (Table 2). However, there was no significant or clinically relevant difference in the prevalence of vaccine-preventable types. It should be noted that overall, 1.7% of the AMPLICOR positives could not be confirmed by LA and the in-house assay, hence these were deemed as negative on genotyping.

**Table 2:** Crude and age-adjusted cervical human papillomavirus (HPV) DNA prevalence and prevalence of multiple HPV infections by Indigenous status (note '/' denotes 'and/or')^a^

HPV Prevalence	Non-Indigenous, 15 to 40 years of age (n = 1494)	Indigenous, 15 to 40 years of age (n = 655)	
	**Age-adjusted^b ^HPV prevalence**	**Age adjusted to Australian population^a ^**	**Indigenous population^c ^**
HPV16/18	12.8% (10.8 to 14.8)	12.2% (9.7 to 14.6)	13.8% (11.1 to 16.6)
HPV genotypes 6/11/16/18	15.3% (13.3 to 17.4)	14.1% (11.5 to 16.7)	15.9% (13.1 to 18.8)
Cross-protection 16/18/31/45	17.1% (14.9 to 19.3)	15.6% (12.9 to 18.3)	17.4% (14.4 to 20.4)
HR^d ^HPV 13 types^e^	30.0% (27.5 to 32.3)	31.3% (27.7 to 34.9)	33.2% (29.5 to 36.8)
HR HPV 18 types^f^	33.5% (31.0 to 36.0)	35.4% (31.8 to 39.1)	37.5% (33.8 to 41.2)
HR HPV-11 types (non-16/18)	23.7% (21.3 to 26.1)	25.9% (22.4 to 29.3)	27.3% (23.8 to 30.9)
HR HPV-16 types (non-16/18)	28.6% (26.1 to 31.0)	30.6% (27.1 to 34.2)	32.3% (28.6 to 36.0)
Any HPV	41.5% (38.9 to 44.1)	47.5% (43.6 to 51.3)	49.3% (45.5 to 53.2)
LR^g ^HPV	24.8% (22.3 to 27.2)	28.7% (25.2 to 32.2)	30.1% (26.5 to 33.7)
LR only present	8.0% (6.6 to 9.39)	12.0% (9.41 to 14.7)	11.9% (9.3 to 14.5)
HPV genotypes 6/11/16/18 only	3.5% (2.6 to 4.5)	3.5% (2.1 to 5.0)	4.0% (2.3 to 5.6)
Multiple HPV types (crude prevalence)			
Range (min-max)	0 to 13	0 to 10	
None	58.8% (56.3 to 61.3)	50.5% (46.6 to 54.5)	
One	17.5% (15.6 to 19.5)	22.0% (18.8 to 25.3)	
Two	9.1% (7.7 to 10.7)	12.4% (9.9 to 15.1)	
Three	7.0% (5.7 to 8.4)	5.8% (4.1 to 7.9)	
Four or more	7.6% (6.3 to 9.1)	9.3% (7.2 to 11.8)	

^a^Data are % (95% CI exact binomial), unless otherwise stated.

^b^Age-standardized to the Australian female population aged 17 to 40 years, 2005, Australian Bureau of Statistics (NB there were only eight women aged 15 to 16 years.)

^c^Age-standardized to the Australian Indigenous female population aged 17 to 40 years, 2005, unpublished experimental estimates, Australian Bureau of Statistics. Single year of age experimental Indigenous population estimates may be subject to errors that cannot be fully adjusted for in the population estimates compilation process.

^d^HR = high-risk.

^e^HR HPV types 16,18,31,33,35,39,45,51,52,56,58,59,68

^f^As above plus HPV types 26,53,66,73 and 82.

^g^LR = low-risk.

HPV16 was the most common genotype in both Indigenous and non-Indigenous women, with types 51, 52, 18 and 39 being the other four most common high-risk types in both groups (Table 3). There was a significant difference in the prevalence of HPV68 (a high-risk type) between Indigenous and non-Indigenous women (OR = 3.8, 95% CI 1.9 to 7.6; *P *< 0.001), although caution is needed in interpreting this finding given the multiple comparisons of genotype groups performed.

**Table 3:** Human papillomavirus (HPV) types in women ≤40 years by Indigenous status, n (%)

HPV type	Non-Indigenous women (*n *= 1494)	Indigenous women (*n *= 655)
6	47 (3.1%)	13 (2.0%)
11	9 (0.6%)	6 (0.9%)
16^a^	141 (9.4%)	69 (10.5%)
18^a^	62 (4.1%)	25 (3.8%)
2	0	1 (0.2%)
31^a^	56 (3.7%)	22 (3.4%)
33^a^	31 (2.1%)	18 (2.7%)
35^a^	16 (1.1%)	14 (2.1%)
39^a^	58 (3.9%)	24 (3.7%)
40	16 (1.1%)	4 (0.6%)
42	61 (4.1%)	19 (2.9%)
45^a^	23 (1.5%)	15 (2.3%)
51^a^	94 (6.3%)	37 (5.6%)
52^a^	57 (3.8%)	36 (5.5%)
53	82 (5.5%)	34 (5.2%)
54	49 (3.3%)	24 (3.7%)
55	29 (1.9%)	8 (1.2%)
56^a^	39 (2.6%)	16 (2.4%)
58^a^	44 (2.9%)	20 (3.1%)
59^a^	39 (2.6%)	22 (3.4%)
61	56 (3.7%)	33 (5.0%)
62	65 (4.4%)	35 (5.3%)
64	1 (0.1%)	0
66	61 (4.1%)	23 (3.5%)
67	24 (1.6%)	8 (1.2%)
68^a,b^	13 (0.9%)	21 (3.2%)
69	1 (0.1%)	4 (0.6%)
70^b^	21 (1.4%)	28 (4.3%)
71^b^	1 (0.1%)	7 (1.1%)
72^c^	2 (0.1%)	7 (1.1%)
73	40 (2.7%)	21 (3.2%)
81^b^	20 (1.3%)	29 (4.4%)
82 (previously IS39) [29]	20 (1.4%)	10 (1.6%)
83	19 (1.3%)	15 (2.3%)
84	61 (4.1%)	19 (2.9%)
89 (previously CP6108) [29]	68 (4.6%)	31 (4.7%)
No type detected^b^	879 (58.8%)	332 (50.6%)
Multiple	354 (23.7%)	180 (27.4%)
16/18	185 (12.5%)	86 (13.1%)
6/11/16/18	220 (14.7%)	102 (15.5%)

16/18 only	44 (2.9%)	21 (3.2%)

6/11/16/18 only	53 (3.5%)	26 (4.0%)

^a^Established high-risk HPV type.

^b^χ^2 ^*P *< 0.001.

^c^χ^2 ^*P *= 0.002.

Among Indigenous women, the high-risk types HPV56 and HPV58 were detected more frequently in women living in remote areas (HPV56 3.6% versus 1.2%, *P *= 0.05; and HPV58 4.5% versus 1.5%, *P = *0.03), whereas the high-risk type HPV59 was found more frequently in women living in non-remote areas (5.2% versus 1.5%, *P *= 0.008). The low-risk (LR) types HPV71 and HPV72 were found in women in remote areas (each in 2.1%; *P *= 0.015). HPV81 was also more common among women in remote areas (6.1% versus 2.8%; *P *= 0.04). Again, caution is needed in interpreting these findings, because of the multiple comparisons.

### Association of age with HPV detection

**Figure 1:**
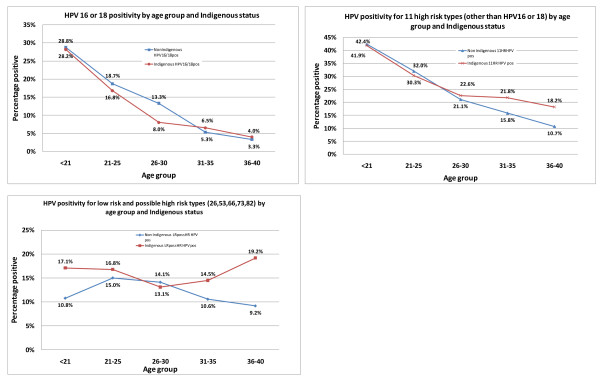
**(A-C): Prevalence of human papilloma virus (HPV) by age group and Indigenous status**. (A) HPV16 or 18; (B) 11 high-risk HPV types without 16/18; (C) low-risk and possible high-risk HPV types.

The prevalence of HPV infection was much higher in younger than older women (Figure 1A). The prevalence of genotypes 16 and 18 was similar for Indigenous and non-Indigenous women in each age group. For other genotypes (HR, probable HR and LR HPV), the prevalence was similar for Indigenous and non-Indigenous women in younger age groups, but higher for Indigenous than non-Indigenous women in the 31 to 35 and 36 to 40 years age groups (Figure 1B, C), with 35% of Indigenous and 22.5% of non-Indigenous 31 to 40-year-old women positive for HPV types other than 16 or 18 (OR = 1.9, 95% CI 1.3 to 2.6, *P *< 0.001). Excluding women with an abnormality on the most recent previous or the current Pap test gave a similar result (Table 4).

**Table 4:** Cervical human papillomavirus (HPV) prevalence^a ^by Indigenous status and age group

Pap status and age group	Indigenous		Non-Indigenous		
	n	%	n	%	*P *value
All women					
15 to 20	117	67.5	139	64.0	0.6
21 to 25	179	56.4	359	54.0	0.6
26 to 30	137	39.4	383	43.6	0.4
31 to 35	124	41.1	341	30.8	0.04
36 to 40	99	39.4	272	22.1	0.001
Women with normal results^b^					
15 to 20	90	61.1	99	52.5	0.2
21 to 25	123	46.3	255	44.7	0.8
26 to 30	105	33.3	323	37.5	0.4
31 to 35	98	34.7	298	25.5	0.08
36 to 40	84	33.3	240	18.3	0.004

^a^Prevalence of any HPV genotype,

^b^Current Pap test and most recent Pap-test results not known to be abnormal.

In the 36 to 40 years age group, 39.4% of Indigenous women had detectable HPV (any HPV genotype) compared with 22.1% of non-Indigenous women (OR = 2.3, *P *< 0.001). The higher HPV prevalence for Indigenous women was partly explained by a higher prevalence of HPV risk factors. In this age group, Indigenous and non-Indigenous women had similar distribution of age by single year (mean age 37.9 versus 38.0, *P *= 0.4, *t*-test) and hormonal contraceptive use (22.4% versus 25.1%, *P *= 0.6), but Indigenous women were more than twice as likely to smoke (45.9% versus 21.8%, *P *< 0.001) and more than seven times as likely to have a current high-grade Pap-test result (3.1% versus 0.4%; *P *= 0.03) or to be having their first ever Pap (n = 3 (3.2%) versus n = 1 (0.4%), *P *= 0.03). Adjusting in multivariate analyses for age, hormonal contraceptive use, smoking, low-grade Pap-test results and most recent previous Pap status reduced, but did not eliminate, the excess risk of HPV detection for Indigenous women (OR = 1.8, 1.03 to 3.2; *P *= 0.04). High-grade results were too infrequent to be included in this model.

#### Detection of multiple HPV types

**Figure 2:**
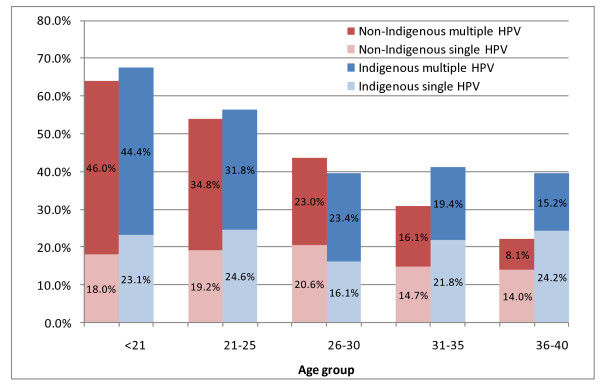
**Percentage of women with single and multiple cervical human papillomavirus (HPV) DNA detection by age group and Indigenous status**.

Overall, 57.6% of HPV-positive non-Indigenous women and 55.6% of HPV-positive Indigenous women had multiple types detected. However, infection with multiple types was less prevalent with increasing age (Figure 2). Although the prevalence of HPV infection was higher for Indigenous than non-Indigenous women in the 36-40 years age group, the proportion of infected women with multiple infections was similar (Indigenous 38.5%, non-Indigenous 36.7%, *P *= 0.9).

In a multivariate analysis of HPV-positive women (including Indigenous status, age, hormonal contraception, smoking, most recent previous Pap status, current Pap status), only younger age (for each year's increase in age: OR = 0.939, 95% CI 0.918 to 0.961, *P *< 0.001) and having a low-grade Pap abnormality on current Pap testing (OR 1.9, 95% CI 1.4 to 2.8, *P *< 0.001) were significantly associated with the detection of multiple HPV types.

#### Predictors of cervical HPV detection

As expected, having a current abnormality on a Pap test was strongly associated with HPV DNA detection (HPV DNA detected in 97.8% of cases with HSIL (OR = 76, *P *< 0.001) and 85.9% LSIL (OR = 10, *P *< 0.001). Having had an abnormality on the previous Pap test was also strongly associated with HPV DNA detection (HPV DNA detected in 70% of cases; OR = 3.4, *P *< 0.001).)

All variables of interest were significantly associated with the detection of any HPV DNA in univariate analysis (Table 5). In multivariate analysis, Indigenous status and hormonal contraceptive use became non-significant. In the stratified analysis, remote area of residence was not a significant predictor of any HPV DNA detection for Indigenous women in either univariate or multivariate analyses (data not shown). However, for non-Indigenous women, residence outside of a major city was associated with a lower risk of any HPV detection (35% versus 46%; OR = 0.6, 95% CI 0.5 to 0.8, *P *< 0.001), which remained significant in multivariate analysis (OR = 0.6, 95 CI 0.5 to 0.8, *P *< 0.001).

**Table 5:** Univariate and multivariate associations with detection of any human papillomavirus (HPV) type, all women aged ≤40 years (n = 2152).

Variable	Any HPV-positive, %	OR (95%CI)	*P *value	Adjusted OR (95%CI)	*P *value
Indigenous^a^					
Yes	49.4%	1.4 (1.2 to 1.7)	< 0.001	1.1 (0.9 to 1.4)	0.3
No	41.2%				
Smoking^b^					
Yes	51.8%	1.6 (1.4 to 2.0)	< 0.001	1.4 (1.2 to 1.8)	< 0.001
No	39.5%				
Age, years^c^		0.915 (0.902 to 0.929)	< 0.001	0.921 (0.908 to 0.935)	< 0.001
Age, 5-year bands					
< 21 years	65.8%	5.3 (3.7 to 7.5)	< 0.001	Modeled as continuous variable as above	
21 to 25 years	54.8%	3.3 (2.5 to 4.4)			
26 to 30 years	42.6%	2.0 (1.5 to 2.7)			
31 to 35 years	33.5%	1.4 (1.0 to1.9)			
36 to 40 years	26.7%	Reference			
HC^d^					
Yes	47.0%	1.25 (1.05 to 1.49)	0.01	1.1 (0.9 to 1.3)	0.3
No	41.5%				

^a^Three missing.

^b^Twenty-one missing.

^c^For each one year increase in age.

^d^Twenty missing. HC = hormonal contraception

In both univariate and multivariate analyses, age, smoking and hormonal contraceptive use were all associated with HPV16/18 detection (Table 6). Indigenous status was not associated with HPV16/18 detection. In stratified analyses including only Indigenous women, HPV16/18 detection was not associated with residence in a remote area, in either univariate or multivariate analyses (data not shown). In the stratified analysis of non-Indigenous women, women residing outside of major cities were significantly less likely to have HPV16/18 detected (9.7% versus 14.3%, *P *= 0.001); this variable remained significant in the adjusted analysis (OR = 0.6, 95 CI 0.5 to 0.9, *P *= 0.01).

**Table 6:** Univariate and multivariate associations with detection of human papillomavirus (HPV) types 16 or 18, all women ≤40 years (n = 2152)

Variable	Any HPV-positive, %	OR (95%CI)	*P *value	Adjusted OR (95%CI)	*P *value
Indigenous^a^					
Yes	13.1%	1.1 (0.8 to 1.4)	0.64	0.8 (0.6 to 1.1)	0.2
No	12.4%				
Smoking^b^					
Yes	16.0%	1.6 (1.2 to 2.0)	0.001	1.4 (1.1 to 1.9)	0.01
No	10.9%				
Age, years^c^	-	0.879 (0.858 to 0.900)	< 0.001	0.884 (0.862 to 0.906)	< 0.001
Age, 5-year bands	28.4%	10.9 (5.9 to 20)	< 0.001	Modeled as continuous variable as above	
< 21 years	18.0%	6.1 (3.3 to 11)			
21 to 25 years	12.1%	3.8 (2.1 to 7.0)			
26 to 30 years	5.6%	1.6 (0.8 to 3.2)			
31 to 35 years	3.5%	Ref			
HC^d^					
Yes	17.4%	2.0 (1.5 to 2.6)	< 0.001	1.7 (1.3 to 2.2)	< 0.001
No	9.6%				

^a^Three missing.

^b^Twenty-one missing.

^c^For each one year increase in age.

^d^Twenty missing. HC = hormonal contraception

## Discussion

This study of Australian women presenting for routine Pap testing prior to the introduction of the National HPV Vaccination Program found HPV16 to be the most prevalent HPV genotype in Indigenous and non-Indigenous Australian women equally, and that the five most common high-risk types were the same in both groups. Detection of any HPV genotype was strongly associated with smoking, Pap-test abnormalities and younger age. Indigenous women living in remote areas did not have more HPV genotypes detected, or more or fewer vaccine-preventable types, but for non-Indigenous women, living in a major city was associated with a higher probability of having HPV detected, and for that type to be 16 or 18. Marked differences in the prevalence of HPV between Indigenous and non-Indigenous women were not found, although older Indigenous women (31 to 40 years old) had more non-HPV 16/18 types detected. The dominance of HPV16 is consistent with worldwide prevalence studies, but HPV51, the second most common high-risk type we detected, does not have such a high prevalence globally [15].

It is unclear why Indigenous women in their 30s had higher rates of non-HPV16/18 genotype carriage. Indigenous Australian women have a different demographic profile to non-Indigenous women, as also reflected in our recruited sample, in ways which could have an effect upon their likelihood of HPV infection, that is, younger age, more than twice as likely to smoke, and a higher fertility rate[19]. Indigenous women also have higher rates of other sexually transmitted infections (STIs) [10], and an earlier age at first sexual intercourse[30]. More Indigenous women also live in poverty [19,31], and greater persistence of HPV due to failure of immunological control could be in part attributable to the effects of lower socioeconomic status or general health status, in combination with high rates of smoking, earlier and more pregnancies, and the presence of other STIs. It could also reflect poorer access to cervical cytology screening by Indigenous women in this age group: this has been hypothesized to be an important factor in the higher rates of cervical cancer incidence and mortality observed in Indigenous women [21,32]. Franceschi *et al*. previously found that lower education levels (as a proxy for socioeconomic status) are correlated with cervical cancer risk but not increased HPV prevalence, and may be mediated through risk factors such as earlier age at first intercourse, and higher and earlier parity [33]. Regardless of the underlying reasons, the presence of other high-risk HPV DNA types among women in their 30s clearly emphasizes the need for participation in cervical screening, whether or not a woman has received HPV vaccination. It should be noted that for this population up to 40 years, the Indigenous women had a U-shaped curve of HPV prevalence. This has also been described in other populations, with hypotheses for its occurrence including changes in sexual behaviour with age, reactivation of latent infection and a cohort effect [34].

The rates of absolute HPV prevalence, frequent occurrence of multiple HPV types and high rates of HPV DNA detected in young women are all consistent with other studies of screening populations in western countries using similar highly sensitive assays (as opposed to clinically adapted assays such as Hybrid Capture 2) [17,35-37].

There are three main limitations to our study. Firstly, we did not collect a sexual or reproductive history from participants. This means we cannot use this established predictor to determine whether women recruited were more or less likely than other Australian women to have HPV. Clearly, however, all women participating in the study were sexually active, and should have been so for at least two years, given that screening guidelines in Australia recommended Pap testing commence at the age of 18 years or 2 years after first sexual intercourse, whichever is later. Secondly, because our main focus was to recruit adequate numbers of women in remote area and of Indigenous women, our sample is not geographically or demographically representative of all Australian women. Non-Indigenous women attending free community health services are likely to be of lower socioeconomic status on average than other Australian women. However, the results were stratified by Indigenous status and ARIA status within the two groups of women, with similar recruitment of health services for both Indigenous and non-Indigenous women, allowing for comparison between the two groups. The non-Indigenous women we enrolled had smoking rates similar to those of the women in the general Australian population (in 2004/5, 27% of women aged 25 to 35 years were smokers) [38], similar rates of oral contraceptive (OC) use (63% and 32% of sexually active women aged 20 to 29 and 30 to 39 years, respectively, used OCs in 2001) [39] and similar rates of Pap abnormalities. Lastly, 38 women recruited to our study (1.8%) were eligible because of their age (aged up to 26 years) at the time of recruitment to have received government-funded HPV vaccination. We did not know their HPV vaccination status, but only six of these women could theoretically have finished a complete HPV vaccine course (4 months after 1 July) at the time of Pap collection. These small numbers of vaccine-eligible women could not influence the study findings.

A truly population-based HPV DNA prevalence survey of women pre-vaccination in Australia was not feasible for several reasons. A random sample of women screened in the national program was not possible because the national program uses conventional dried slides rather than LBC. Under Australian legislation, the slide must be retained for a minimum of 10 years, but testing for HPV involves destruction of the slide. Women who choose to have LBC in Australia have to pay privately for the test, and are not therefore a representative sample. Australia currently has no national health survey with a biological sample collection component (for example, urine sample or vaginal swab) which could otherwise form a sample frame for HPV testing of other suitable (although probably less sensitive) sample types, as has been conducted in the UK (urine) and USA (vaginal swab)[40]. Although the first such survey with a biological sampling component is planned for 2011, it is explicitly stated that no STI testing is permissible within the survey framework [41].

Taken together with baseline HPV seroprevalence data for Australia,[42] the WHINURS study data provides an important baseline data set for Australia pre-vaccination. Consistent with WHO recommendations for post-vaccination surveillance systems [43], studies are underway to use WHINURS sites as sentinel sites for post-vaccination surveillance, with identical recruitment methods, sample collection and typing methods used, to allow comparison between pre- and post-vaccination type-specific HPV prevalence in young women presenting for Pap testing.

## Conclusions

In conclusion, we found no evidence that HPV16/18 prevalence differs significantly for Australian women according to their Indigenous status or, for Indigenous women, whether they lived in remote areas. However, we did identify differences in risk-factor prevalence and in the prevalence of some other HPV genotypes. Indigenous Australian women currently have higher rates of cervical cancer, probably due to factors such as smoking, higher fertility and lower rates of participation in cervical screening. This reinforces the importance of cervical screening as a complement to vaccination for all women, and the value of baseline data on HPV genotype prevalence by Indigenous status and residence for monitoring of the effect of a vaccine program.

## Competing interests

Other than the grant funding received for this study (see below), JC, ST, MS and DS have no competing interests. JB and PM were co-authors on an investigator-driven HPV serosurveillance study in which laboratory testing was funded by CSL Limited. JB and SG are partner investigators on an Australian Research Council linkage grant in which CSL is a partner investigator. SG has received advisory board fees and grant support from CSL and GlaxoSmithKline, and lecture and consultancy fees from Merck and Co. SG reports having previously owned stock in CSL. SG has received grant support through her institution from Merck and Co and GlaxoSmithKline (GSK) to carry out clinical trials for HPV/cervical cancer vaccines, and she is a member of the Merck global advisory and scientific advisory boards.

## Authors' contributions

SG, JB and PM designed the study with assistance from JC and DS. SG coordinated the study, and managed recruitment of participating sites and the study team, including performing study workshops at most sites, and was responsible for overall specimen and laboratory methods. JC coordinated site recruitment in the Northern Territory, and DS coordinated site recruitment in Western Australia. ST managed specimen coordination and HPV DNA typing which was performed by MS. JB analyzed the study data, with assistance from JC, PM and MS. Data interpretation was led by JB, JC, PM and SG. SG and JB drafted the initial manuscript with critical revisions by all authors. All authors approved the final version.

## Pre-publication history

The pre-publication history for this paper can be accessed here:

http://www.biomedcentral.com/1741-7015/9/104/prepub
